# Dynamics of Redox Metabolism during Complete Metamorphosis of Insects: Insights from the Sunflower Caterpillar *Chlosyne lacinia* (Lepidoptera)

**DOI:** 10.3390/antiox13080959

**Published:** 2024-08-07

**Authors:** Daniel C. Moreira, Marcelo Hermes-Lima

**Affiliations:** 1 Research Center in Morphology and Applied Immunology, Faculty of Medicine, University of Brasilia, Brasilia 70910-900, Brazil; 2 Cell Biology Department, Biological Sciences Institute, University of Brasilia, Brasilia 70910-900, Brazil; hermes.unb@gmail.com

**Keywords:** antioxidant, glutathione, hypoxia, oxidative stress, reactive oxygen species

## Abstract

Complete insect metamorphosis requires substantial metabolic and physiological adjustments. Although oxidative stress has been implicated in metamorphosis, details on redox metabolism during larva-to-pupa and pupa-to-adult remain scarce. This study explores redox metabolism during metamorphosis of a lepidopteran (*Chlosyne lacinia*), focusing on core metabolism, antioxidant systems and oxidative stress. The larva-to-pupa transition was characterized by increased lactate dehydrogenase and glutathione peroxidase (GPX) activities, coupled with depletion of reduced glutathione (GSH), high disulfide-to-total-glutathione ratio (GSSG/tGSH), and increased lipid peroxidation. As metamorphosis progressed, metabolic enzyme activities, citrate synthase and glucose 6-phosphate dehydrogenase increased, indicating heightened oxidative metabolism associated with adult development. Concurrently, GSH and GPX levels returned to larval levels and GSSG/tGSH reached its most reduced state right before adult emergence. Adult emergence was marked by a further increase in oxidative metabolism, accompanied by redox imbalance and enhanced antioxidant mechanisms. These findings highlight a fluctuation in redox balance throughout metamorphosis, with periods of oxidative eustress followed by compensatory antioxidant responses. This study is the first to identify concurrent changes in metabolism, antioxidants, redox balance and oxidative stress throughout metamorphosis. Our findings extend knowledge on redox metabolism adjustments and highlight redox adaptations and oxidative stress as natural components of complete insect metamorphosis.

## 1. Introduction

The complete metamorphosis of insects is a fascinating process that remarkably transforms larvae into adults. Adults display structures that are either absent or underdeveloped in their immature stages [1]. These structures develop during the pupal stage, where larval tissues are repurposed and remodeled to form new structures [2,3]. This process encompasses cell proliferation, differentiation, and cell death as part of tissue remodeling [4]. Pupal energy needs are met by reserves accumulated during the larval stage, as pupae typically do not feed [5]. Consequently, on the top of the extensive transformation, metamorphosis requires significant metabolic and physiological adjustments, including changes in energy metabolism and nutrient utilization [4,6,7]. Contrary to any expectation of high metabolic activity during the larva-to-adult transition due to intense tissue reorganization, pupal metabolism in major holometabolous insect groups (the ‘big four’: Diptera [8], Lepidoptera [9], Coleoptera [9], and Hymenoptera [10]) exhibits a U-shaped pattern. Metabolic rates are initially high at pupation, decrease during metamorphosis, remain low for a period, and then increase as adult emergence nears. This is the pattern of different indicators of metabolism, including oxygen consumption [11], carbon dioxide production [8], activity of enzymes involved in oxidative metabolism [8], and the rate of amino acid incorporation [12].

As signaling molecules, reactive species (RS), such as free radicals, reactive oxygen species, reactive nitrogen species, and reactive sulfur species, are implicated in key cellular processes like proliferation, differentiation, tissue remodeling, and metabolism [13,14,15]. These molecular species are continuously produced through various cellular enzymatic and non-enzymatic reactions [16] and are neutralized by antioxidant systems, maintaining a physiological redox steady state [17]. The interplay between antioxidants and RS is part of a broader reactive species interactome—a network formed by RS, antioxidants, redox-sensitive molecules, and downstream targets that regulate cellular redox signaling [18]. Through the redox code [19], compensatory mechanisms are activated to re-establish balance when the redox steady state is disturbed [20]. This adaptive response, termed oxidative eustress [21], typically involves mounting antioxidant systems to counteract a shift towards a more oxidized state. In some situations, the redox balance shifts away from its setpoint to the extent that signaling and control are compromised. This deviation, called oxidative distress, is characterized by excessive oxidation, disrupted cellular signaling, and the loss of function of biomolecules [22].

Fluctuations in oxygen availability and consumption often lead to redox imbalance, thereby eliciting oxidative stress [23,24,25]. For example, in rabbit hearts, the reestablishment of oxygen supply after a period of oxygen deprivation (post-ischemic reperfusion) dramatically increases ROS production [25] and reduces cell viability by 90% [24]. This contrasts to the remarkable tolerance of other animal species that naturally experience drastic variations in metabolic rate or environmental oxygen levels as part of their natural life histories [26]. In most cases, these animals allocate resources in building a cellular stress response that includes the activation antioxidant systems upon hypoxia [27]. This was originally interpreted as an anticipatory response, termed ‘Preparation for Oxidative Stress’ [28], to deal with the expected overproduction of RS upon reoxygenation. It is now recognized that this activation is actually an oxidative eustress in which a moderate oxidant stimulus triggers adjustments in redox systems [29], which would ultimately prevent or minimize oxidative distress when metabolic rates return to normal during arousal or reoxygenation.

The intricate relationship between energy metabolism and redox reactions is challenging to disentangle. This is illustrated by the several sites of RS production along the mitochondrial electron transport chain [30]. Moreover, NADPH, a product of metabolism, can be directed towards either the generation of RS (via NADPH oxidases) or the regulation of RS levels (through glutathione and thioredoxin systems). As such, redox signaling senses, adapts to and communicates changes in metabolism [31]. Consequently, numerous studies have explored how redox metabolism is influenced by or influences substantial shifts in metabolic rate, such as diapause [32,33], estivation [34,35,36], and hibernation [37,38,39], often observing the activation of endogenous antioxidants [29,40]. On the other hand, the metamorphosis of holometabolous insects is a phenomenon characterized by a decline and recovery of metabolism that, despite resembling other metabolic suppression situations, remains underexplored from a redox perspective.

Although the pupal phase is critical for the formation of the adult insect, research on redox metabolism during the ontogeny of holometabolous insects mainly focuses on the larval stage [41,42,43,44,45]. Studies encompassing the pupal phase often limit their scope to a single [43,44,46,47,48,49] or a couple of time points (typically early and late pupa) [50,51,52] to represent this highly dynamic developmental stage. Such approaches potentially overlook biochemical transformations occurring throughout the pupal phase. Moreover, the few studies that do examine redox metabolism changes during the pupal stage of holometabolous insects typically restrict their focus to a single antioxidant system, such as SODs [53,54]. For example, the expression levels of SOD1 and SOD2 were monitored from the fifth larval instar to the adult in the silkworm *Bombyx mori*, revealing that both SOD forms are downregulated as the animal approaches pupation [53]. In the fruit fly *Drosophila melanogaster*, SOD activity follows a U-shaped curve from eggs to adults [55]. Still, the occurrence of other compensatory responses of antioxidant systems and actual effects on the redox balance and oxidative stress remain unknown. Consequently, these studies offer a limited perspective of the redox adaptations associated with insect metamorphosis, especially the dynamics of redox metabolism during the formation of the adult insect, from pupation to adult emergence.

The present study set out to shed light on these dynamics by investigating changes in the redox metabolism during the metamorphosis of a holometabolous insect. Using the bordered patch *Chlosyne lacinia* (Lepidoptera: Nymphalidae) as a model, we sought to address the hypothesis that antioxidant systems are differentially regulated throughout metamorphosis, adjusting to the changing redox demands of transitioning developmental stages. We have previously identified key biochemical adjustments of enzymes associated with redox metabolism in the diapause of *C. lacinia* [32]. Namely, the activities of glutathione transferase and the NADPH-generating isocitrate dehydrogenase are 4.7-fold and 7.6-fold higher at the beginning of diapause than those in active animals in the same instar [32]. Here, we predicted that alterations in the redox balance will correlate with shifts in energy metabolism, with changes in metabolism influencing redox states. To test these hypotheses, we measured the activities of key metabolic and antioxidant enzymes, as well as oxidative stress markers, at multiple time points spanning from the final larval instar to the adult stage of *C. lacinia*. By doing so, we aimed to address the questions: How do antioxidant systems behave during metamorphosis? Are there actual alterations in the redox balance throughout this transformative process? And does metamorphosis in holometabolous insects involve the activation of endogenous antioxidants as observed in natural situations of metabolic rate suppression? Our interest on *C. lacinia* stems from its status as one of the main pests defoliating sunflower crops [56], and its remarkable resilience to the severe dry seasons of the Brazilian tropical savanna (Cerrado ecoregion), when they enter diapause and remain viable for several months until the rainy season arrives [32]. We have previously documented its biochemical adjustments during larval diapause [32], and here we aim to study redox dynamics and metabolism during its normal development, throughout its complete metamorphosis.

## 2. Methods

### 2.1. Animals

Eggs of the sunflower caterpillar, *Chlosyne lacinia* (Lepidoptera: Nymphalidae), were collected in Cerrado areas (15°43′ S, 47°53′ W). Eggs and posterior development stages were maintained at 25 °C, 55 ± 10% relative humidity, and 12:12 light–dark cycles as previously described [32,57]. After egg hatching, larvae were fed daily with fresh leaves of *Tithonia diversifolia*. Animals were kept under these conditions until sampled for analysis at different developmental stages. Last instar wandering larvae (L); pupae at days 0, 1, 2, 3, 4, 5, 6 after pupation; and newly-emerged adults (A) were frozen in liquid nitrogen and stored at −80 °C until assayed. Animals were inspected daily, and newly formed pupae (day 0 group) were identified by their characteristic color and soft exoskeleton. The day 0 group consisted of animals that had pupated within the previous 24 h. For all variables, the observational unit was a single animal. For the measurement of enzymatic activities and glutathione, five animals were assessed per group (*N* = 5), with the exception of a few groups detailed in the figure legends where *N* = 4. For the measurement of lipid peroxidation, six animals were assessed per group (*N* = 6), except for the larvae group, where *N* = 5. Details on the exact sample size for each biochemical measurement can be found in the Supplementary Materials. Animal collection, housing and experimentation were registered and authorized by the Chico Mendes Institute for Biodiversity Conservation, Brazilian Ministry of the Environment (SISBIO 63338-1, SISGEN A171123).

The lifespan of *C. lacinia* varies from 35 to 45 days when observed in natural outdoor conditions [58]. When reared in ideal laboratory conditions, the embryonic phase lasts about 6 days, the larval stage around 19 days, pupation approximately 6 days, and adults live for roughly 7 days [57]. Even under optimal laboratory conditions, about 30% of the larval population goes into diapause at the third instar, a period of dormancy lasting from one to six months [32,57].

### 2.2. Enzymatic Activity

Whole animals were homogenized in 50 mM phosphate buffer (pH 7.2) containing 0.5 mM EDTA, 1 mM phenylmethylsulfonyl fluoride and 1:1000 (*v*/*v*) protease inhibitors cocktail (P8340, Sigma-Aldrich, St. Louis, MO, USA) using a tissue master 125 homogenizer (OMNI, Kennesaw, GA, USA). The homogenates were centrifuged at 15,000× *g* for 15 min at 4 °C. The supernatant was transferred to new tubes and kept on ice until the measurement of enzymatic activity, which occurred shortly after centrifugation. Enzymatic activities were measured using colorimetric kinetic assays previously described [32,38] and briefly outlined below.

The capacity of oxidative metabolism was assessed by measuring the activity of citrate synthase (CS), which catalyzes the first step of the tricarboxylic acid cycle. The assay reaction medium was composed of 0.5 mM oxaloacetic acid, 0.1 mM 5,5′-dithio-bis-(2-nitrobenzoic acid) (DTNB), and 0.06 mM acetyl-CoA. Additionally, the activity of glucose 6-phosphate dehydrogenase (G6PD) served as an indicator of the ability to generate NADPH via the pentose phosphate pathway, essential for supplying reducing potential to antioxidant systems. The assay reaction medium was composed of 5 mM MgCl_2_, 0.2 mM NADP^+^, and 1 mM glucose 6-phosphate. We also assessed the capacity for anaerobic ATP production by measuring lactate dehydrogenase activity. The assay reaction medium contained 1 mM pyruvate and 0.2 mM NADH. These measurements provide insights into the pathways supporting oxidative metabolism, NADPH production, and anaerobic energy generation.

To evaluate the capacity for controlling the levels of H_2_O_2_ and other hydroperoxides, which are important redox signaling agents, the activities of catalase and glutathione peroxidase (GPX) were measured. Catalase activity was assessed in a reaction medium containing 10 mM hydrogen peroxide. For GPX, the assay medium contained 4 mM NaN_3_, 5 mM reduced glutathione (GSH), 0.1 U/mL glutathione reductase, 0.2 mM NADPH, and 0.2 mM cumene hydroperoxide. Glutathione transferases (GSTs) constitute another important component of antioxidant systems. This family of enzymes functions by conjugating electrophilic xenobiotics, as well as products of oxidative damage to lipids, facilitating their elimination. The assay medium for measuring GST activity contained 1 mM GSH and 1 mM 1-chloro-2,4-dinitrobenzene. Moreover, we attempted to measure the activity of glutathione reductase (GR), an enzyme crucial for recycling disulfide glutathione back to its reduced form, thereby supporting the antioxidant capacity of cells. However, in homogenates from *C. lacinia*, GR activity was below the detection limit of the employed method [59], as previously reported for *C. lacinia* larvae [32].

All assays were buffered with 50 mM potassium phosphate buffer containing 0.5 mM EDTA at pH 7.2 (for metabolic enzymes) or 7.0 (for antioxidant enzymes). For all enzymes, control assays were run in parallel in the absence of (i) homogenate and (ii) substrate(s) (one or both), whose rates were subtracted from the rates of the complete assay, correcting for changes independent of enzyme activity. Enzymatic activities are expressed as per milligram of soluble protein in the supernatants measured with Coomassie Brilliant Blue G-250 [60].

### 2.3. Glutathione and Oxidative Stress Markers

Oxidative damage to membrane polyunsaturated fatty acids, through lipid peroxidation, disrupts membrane integrity and leads to several adverse effects [61]. This process results in elevated levels of lipid peroxidation end-products, such as malondialdehyde (MDA) and 4-hydroxynonenal, whose levels rise under oxidative stress [62]. The thiobarbituric acid reactive substances (TBARS) assay, which quantifies lipid peroxidation based on the interaction between MDA and thiobarbituric acid (TBA), serves as a cheap, straightforward and widely used general measure of lipid peroxidation and oxidative stress [62,63]. In this study, whole animals were homogenized in 10% (w/v) trichloroacetic acid (TCA) using a Tenbroek glass homogenizer. The crude homogenate was split into two aliquots: one for the measurement of TBARS levels [64,65] and another for the measurement of glutathione (described below). Briefly, the crude homogenate was mixed with a reaction medium (10% TCA; 0.25% TBA, 0.17 M HCl, and 0.3 mM butylated hydroxytoluene). For a control, a similar setup omitted TBA. After heating in boiling water (95 °C) for 15 min and cooling at room temperature, the mixtures were centrifuged at 10,000× *g* for 6 min at 4 °C. The resulting supernatants were then transferred to 96-well plates and measured at wavelengths of 532 and 600 nm in a microplate reader.

The remaining homogenate aliquot was centrifuged at 10,000× *g* for 10 min at 4 °C. The supernatant was transferred to a new tube and kept on ice until the measurement of total glutathione (tGSH) and disulfide glutathione (GSSG), which occurred shortly after centrifugation, using the enzymatic recycling method [65,66]. Briefly, supernatant aliquots were combined with a reaction mixture composed of 100 mM potassium phosphate (pH 7.0), 1 mM EDTA, 0.238% TCA, 0.48% ethanol, 0.1 mM NADPH, 0.1 mM 5,5′-dithiobis(2-nitrobenzoic acid) (DTNB), and 0.05–0.4 U/mL GR from baker’s yeast. This assay relies on the production of 2-nitro-5-thiobenzoate (TNB), detectable at 412 nm, catalyzed by GR in the presence of GSH and DTNB. The rate of TNB production, monitored at 412 nm in the kinetic mode of a microplate reader, correlates with the GSH concentration in the mixture. For exclusive GSSG quantification, samples were pre-treated with 2-vinylpyridine for 1 h prior to the assay. Levels of glutathione in its reduced and disulfide forms were derived by comparing against standard curves spanning 0.025 to 1.5 μM. The GSSG/tGSH ratio and TBARS levels are well-known markers of redox imbalance and oxidative stress [62,67].

### 2.4. Statistics

The data from all experiments were subjected to normality testing using the Shapiro–Wilk test. Subsequently, data with parametric distribution were analyzed using one-way ANOVA followed by Tukey’s multiple comparisons test, while data with non-parametric distribution (only G6PD activity) were analyzed using Kruskal–Wallis one-way analysis of variance, followed by Dunn’s multiple comparisons test. In all analyses, statistical significance was defined as *p* < 0.05. Statistical analyses and figure preparation were performed using RStudio version 2021.9.0.351 [68].

## 3. Results

**Figure 1:**
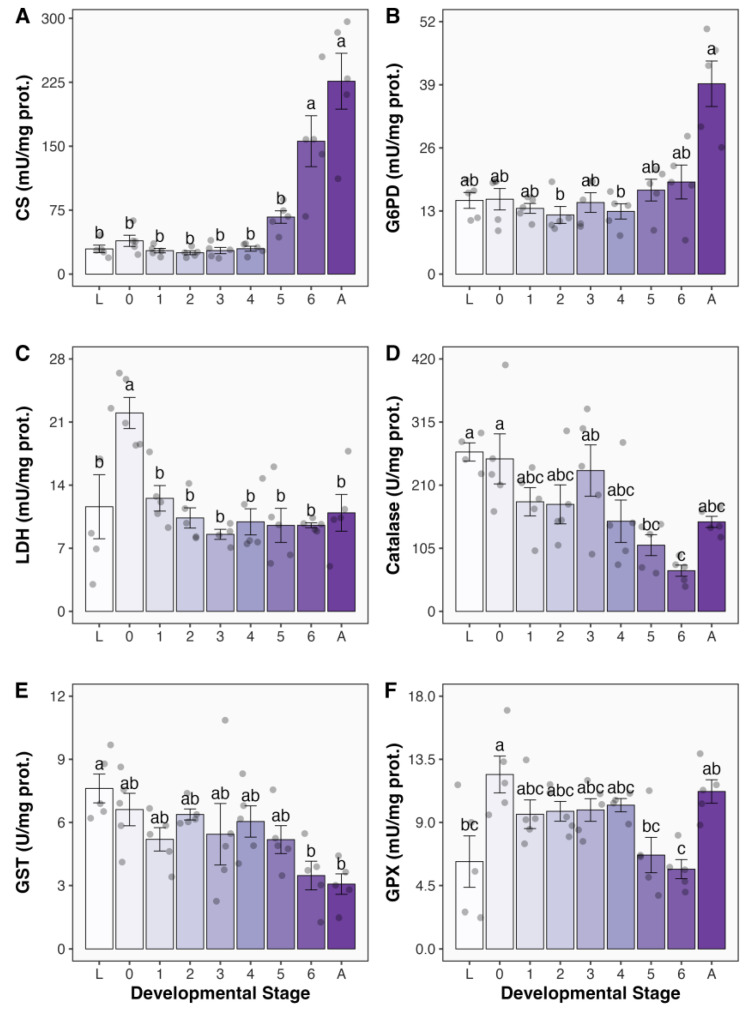
Activities of metabolic and antioxidant enzymes in *Chlosyne lacinia* (Lepidoptera: Nymphalidae) during holometabolous metamorphosis from last instar wandering larvae to adult emergence. (**A**) Citrate synthase (CS) activity, (**B**) glucose 6-phosphate activity dehydrogenase (G6PD) activity, (**C**) lactate dehydrogenase (LDH) activity, (**D**) catalase activity, (**E**) glutathione transferase (GST) activity, and (**F**) glutathione peroxidase (GPX) activity measured in whole body homogenates. Developmental stages are as follows: L, last instar wandering larvae; 0, <24 h since pupation; 1–6, pupae formed for 1–6 days; A, adults emerged for less than 24 h. Compact letters display indicates that groups not sharing lower case letters are significantly different from each other (*p* < 0.05). *N* = 5 for all groups except for LDH in pupae 3 (3), catalase in larvae (L), and GPX in pupae 4 (4), where *N* = 4. Filled circles denote individual data points.

The activities of citrate synthase (CS; Figure 1A) and glucose 6-phosphate dehydrogenase (G6PD; Figure 1B) followed a similar pattern as metamorphosis progressed. They remained relatively stable during the initial days of the pupal stage and increased as adults developed. CS activity increased significantly by 133% on the sixth day when compared with the previous day, indicating that the increases in oxidative metabolism precede adult emergence (Figure 1A). In the case of G6PD, the activity remained around 15.0 mU/mg prot. throughout the pupal phase and increased sharply in emerged adults. In newly emerged adults, G6PD levels were 39.2 mU/mg prot., whereas they were 19.0 mU/mg prot. in the previous development stage. Despite being 2.1-fold, this difference was not statistically significant (Kruskal–Wallis + Dunn’s multiple comparisons test), which can be attributed to the increased variability among individuals that had just emerged to adult life (the activity was, however, significantly 3.0-fold higher than that recorded on the fourth day after pupation). The activity of lactate dehydrogenase (LDH) increased significantly in newly formed pupae, with LDH activity being 90% higher than that in larvae (Figure 1C). However, 24 h after pupation, LDH activity returned to its baseline levels (12.5 mU/mg prot.) and was maintained at approximately 9.8 mU/mg prot. throughout metamorphosis. This finding suggests a transient increase in anaerobic metabolism capacity during the first hours after pupa formation.

Catalase activity gradually decreased as metamorphosis advanced from larvae (265.2 U/mg prot.) to six-day-old pupae (67.7 U/mg prot.), at which point it reached its lowest values, being 26% of that observed in larvae (Figure 1D). In newly emerged adults, catalase activity was 148.8 mU/mg prot., but not significantly different from any experimental group. The activity of glutathione transferase (GST) also gradually declined as metamorphosis progressed (Figure 1E). Once metamorphosis was complete, GST activity in adults (3.1 U/mg prot.) had decreased to 40% of the level observed in larvae (7.6 U/mg prot.). Similar to LDH activity, glutathione peroxidase (GPX) activity doubled in newly formed pupae (12.4 mU/mg prot.) compared to larvae (6.2 mU/mg prot.; Figure 1F). Subsequently, GPX activity remained stable at approximately 9.9 mU/mg prot. until the fifth day after pupae formation, when it slightly decreased to 6.7 mU/mg prot. On day 6, pupae had the lowest level of GPX activity (5.7 mU/mg prot.), which significantly increased by 2-fold when adults emerged (11.2 mU/mg prot.; Figure 1F).

**Figure 2:**
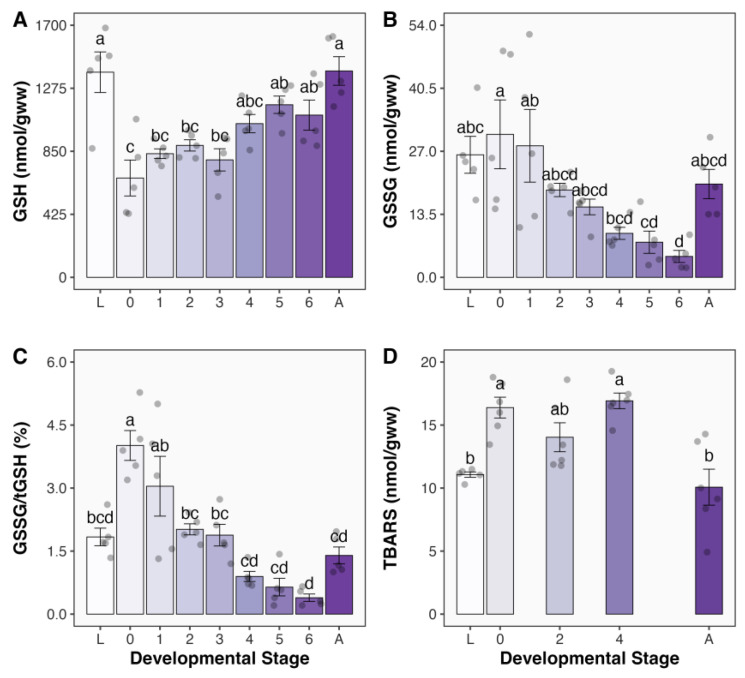
Levels of glutathione and oxidative stress markers in *Chlosyne lacinia* (Lepidoptera: Nymphalidae) during holometabolous metamorphosis from last instar wandering larvae to adult emergence. (**A**) Reduced glutathione (GSH) levels, (**B**) disulfide glutathione (GSSG) levels, (**C**) disulfide to total glutathione (GSSG/tGSH) ratio, and (**D**) thiobarbituric acid reactive substance (TBARS) levels measured in whole body homogenates. Developmental stages are as follows: L, last instar wandering larvae; 0, <24 h since pupation; 1–6, pupae formed for 1–6 days; A, adults emerged for less than 24 h. Filled circles denote individual data points. Compact letters display indicates that groups not sharing lower case letters are significantly different from each other (*p* < 0.05). *N* = 5 for GSH, GSSG, and GSSG/tGSH in all groups. *N* = 6 for TBARS in all groups, except for larvae where *N* = 5. Filled circles denote individual data points.

The levels of total glutathione (tGSH) significantly declined in newly formed pupae (731.1 nmol/gww), being 49% lower than the levels observed in larvae (1436.9 nmol/gww; Supplementary Materials). As metamorphosis progressed, tGSH concentration gradually increased and returned to the levels observed in larvae upon adult emergence (1431.6 nmol/gww; Supplementary Materials). These changes in tGSH were primarily driven by variations in reduced glutathione (GSH) levels, which followed a similar pattern (Figure 2A). GSH levels significantly decreased by 52% in newly formed pupae (669.8 nmol/gww) *versus* larvae (1383.4 nmol/gww) and gradually returned to larval levels at adult emergence (1391.7 nmol/gww).

Conversely, the levels of disulfide glutathione (GSSG) remained stable during the initial 24 h after pupation (26.2 nmol/gww in larvae and 30.6 nmol/gww in day 0 pupae; Figure 2B). Then, it exhibited a stepwise tendency to decrease until the sixth day from pupae formation, when GSSG levels were the lowest at 4.5 nmol/gww, being 83% lower than those observed in larvae (Figure 2B). Later, GSSG levels returned to baseline levels in newly emerged adults (20.0 nmol/gww). In newly formed pupae, the GSSG/tGSH ratio significantly increased by 2.2-fold compared to that in larvae (Figure 2C). It then gradually decreased, reaching the lowest level on the sixth day after pupation, when GSSG/tGSH ratio was only 21% of that in larvae. At adult emergence, GSSG/tGSH ratio increased from 0.4% in six-day-old pupae to 1.4% in adults, but this difference was not statistically significant. These findings indicate the occurrence of a transient redox imbalance at pupation and a shift towards a more oxidized state at adult emergence.

The levels of TBARS increased significantly by 48% from 11.1 nmol/gww in larvae to 16.4 nmol/gww in newly formed pupae and remained elevated compared to those recorded in larvae as metamorphosis progressed (Figure 2D). When adults emerged, their TBARS levels (10.1 nmol/gww) were similar to those found in larvae (Figure 2D), indicating that the oxidative stress at pupation was resolved by the time of adult emergence.

## 4. Discussion

**Figure 3:**
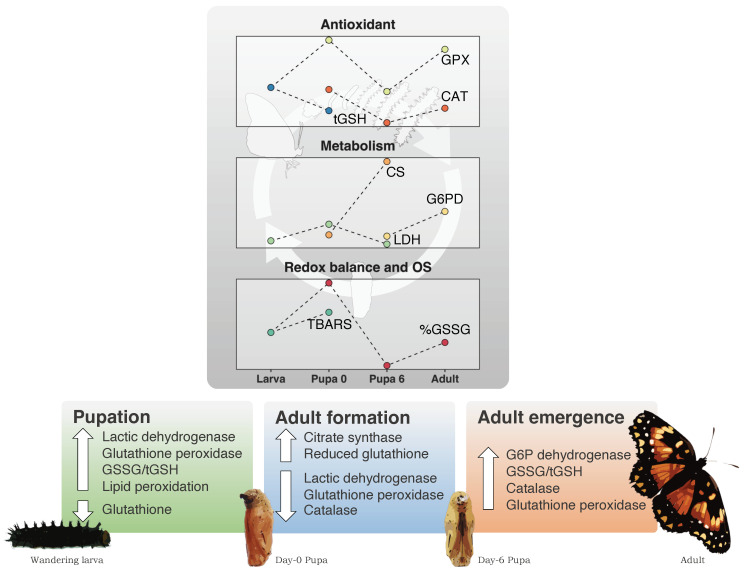
Main biochemical events in three key moments of complete metamorphosis in *Chlosyne lacinia* (Lepidoptera: Nymphalidae): pupation, adult formation during the pupal phase, and adult emergence. Pupation is associated with increased anaerobic metabolism capacity, glutathione depletion, redox imbalance, oxidative stress and upregulation of glutathione peroxidase (GPX). As adult tissues are formed during the pupal stage, aerobic metabolism capacity increases, glutathione levels are restored and the activity of catalase and GPX decrease. Upon adult emergence, the oxidation of glutathione indicates redox imbalance, which seems to be counter balanced by a higher capacity to produce NADPH and Increased catalase and GPX activities. OS, oxidative stress markers.

The present study aimed to elucidate the biochemical events underlying the formation of the adult of holometabolous insects, specifically by assessing energetic metabolism, antioxidants, redox balance, and oxidative stress throughout this process. We show, for the first time, concurrent changes in energetic metabolism, redox balance, antioxidant systems and oxidative stress during complete insect metamorphosis. Metamorphosis involved substantial biochemical changes, particularly during the larva-to-pupa transition and at adult emergence (summarized in Figure 3). These stages were marked by distinct shifts in enzymatic activities and redox states. The larva-to-pupa transition was characterized by increased activities of LDH and GPX, coupled with a reduction in both tGSH and GSH levels. This stage also exhibited a rise in the GSSG/tGSH ratio and lipid peroxidation, indicating a temporary redox imbalance and oxidative stress. As metamorphosis progressed towards adult emergence, there was a gradual elevation in metabolic enzyme activities, with tGSH, GSH, and GSSG/tGSH levels returning to their values recorded in larvae. Concurrently, the activities of GST, catalase, and GPX, as well as GSSG levels, showed a decreasing trend until the pupal stage that preceded adult emergence. These changes suggest that the initial redox imbalance was addressed, thereby shifting the redox balance towards a more reduced state as metamorphosis progressed. At the stage of adult emergence, however, another event of redox imbalance was evident. Here, there was an increase in the activities of peroxide-detoxifying enzymes and the GSSG/tGSH ratio compared to the preceding pupal stage. Still, this effect was less intense than that observed during the earlier stage of pupation.

The inverted-bell-curve pattern of metabolism during holometabolous metamorphosis, first observed in the mealworm *Tenebrio molitor* by Krogh [69], has been extensively documented across endopterygotes. Activities of core metabolism enzymes and dehydrogenases [70,71], amino acid incorporation and metabolism [12,72], O_2_ consumption [73,74], CO_2_ release [8,9], and heat production [10,75] all converge to a U-shaped metabolic rate curve. Our study revealed a J-shaped trend in the biomarkers of oxidative metabolism in *C. lacinia*. This pattern is similar to those of *Manduca sexta*, *D. melanogaster*, and *T. molitor*, where similar J-shaped curves are observed for mitochondrial cristae surface area, CS activity, and oxygen consumption, respectively [8,9,76]. The apparent deviation of our findings from the typical U-shaped curve may be attributed to the large increase in CS activity as adults near development completion, similar to the pattern observed for mitochondrial content in *M. sexta* [9]. Additionally, changes in whole animal metabolic rate are not necessarily matched by changes at the mitochondrial and biochemical level (i.e., enzyme activity). For example, the increase in mitochondrial content as metamorphosis approaches completion precedes and is not directly proportional to the increase in O_2_ consumption in *M. sexta* [9]. Similarly, the ratio between CO_2_ release and CS activity is not constant during the metamorphosis of *D. melanogaster* [8]. In sum, despite the lack of a typical U-shaped curve, our data on oxidative metabolism align with previous reports on mitochondrial activity measurements during complete metamorphosis of insects.

As metamorphosis progressed and adult emergence approached, we observed an increase in the activity of CS and G6PD, consistent with several insect orders where metabolic rate indicators rise sharply as metamorphosis nears completion. This increase in oxidative metabolism is likely related to mitochondrial biogenesis in adult insect flight muscles [77]. Indeed, the rising part of the U-shaped (or J-shaped) metabolic curve correlates with the substitution of the larval fat body with mitochondria-rich muscle tissue in the thorax of metamorphosing pupae [78]. Given that flight is an energetically demanding type of locomotion [79], it requires high ATP turnover rates [80], particularly in insect flight muscle [81,82]. Hence, insect flight is inherently aerobic [83]. Accordingly, the sharp increase in CS activity, which precedes the rise in metabolic rate, in *D. melanogaster* is attributed to the differentiation of the adult flight muscle [8]. Similarly, here, the development of the flight muscle in *C. lacinia* is probably associated with the observed increase in oxidative metabolism capacity as metamorphosis approaches completion.

Considering that mitochondria are major sources of ROS [30], the increased mitochondrial density and oxidative metabolism in adult insects likely impose oxidative pressure on their antioxidant systems. This assumption is supported by the observed rise in the glutathione disulfide (GSSG) levels and its ratio relative to the total glutathione (tGSH) ratio in adults compared to the preceding developmental stage. Nevertheless, our data indicate effective counteraction of potential oxidative damage by the increase in tGSH levels and activity of enzymes decomposing hydrogen peroxide from day 6 to adult, which is supported by the fall in TBARS levels from pupae to adults. A global increase in the levels of several endogenous antioxidants, including catalase and non-specific peroxidase activities, was observed in active adult specimens of the solitary red mason bee, *Osmia bicornis*, relative to their late pupal stage [50]. The elevation of antioxidant levels in adult stages compared to pupal stages also occurs in other species. Specifically, adult *T. molitor* showed increased activities of catalase and SOD [46], *D. melanogaster* showed increased catalase and SOD activity [55], and *B. mori* showed upregulated expression of several SOD mRNAs [43]. Furthermore, the enhanced capacity to produce NADPH through the pentose phosphate pathway observed here may also play a role in addressing any redox imbalance. Adequate NADPH supply is critical for the reduction of key redox-active molecules, such as GSH and thioredoxin. As such, NADPH is necessary for the functioning of antioxidant systems and contributes to maintaining redox homeostasis and signaling [84,85]. The multiple observations of enhanced antioxidant systems at this stage suggest a conserved biological response across different insect species, where the transition from pupal to adult stages is accompanied by an increase in antioxidant capacity.

Differently from the activity of hydrogen-peroxide decomposing enzymes, GST activity gradually decreased as metamorphosis progressed from larvae to adults. Similarly, in the greater wax moth, *Galleria mellonella*, GST activity declines during metamorphosis and reaches its lowest levels in the late pupal stage and remain low in adults [52]. These observations align with the observation of increased GST family gene expression during larval stages in lepidopterans such as the gypsy moth *Lymantria dispar* [44] and a specific GST gene in the spruce budworm *Choristoneura fumiferana* [86]. Beyond detoxification, GSTs have antioxidant functions [87] and participate in several physiological processes, including hormone synthesis, amino acid catabolism, and cell signaling regulation [88]. Insect GSTs have been extensively investigated for their role in detoxifying xenobiotics, such as insecticides and plant allelochemicals [89,90], which is often linked to host plant adaptation [91]. For instance, exposure to plant-derived chemicals induces GST expression in several lepidopterans, including the common cutworm *Spodoptera litura* [92], *C. fumiferana* [42], *L. dispar* [44], and *G. mellonella* [93]. Taken together, our results and these observations highlight a prominent role of GSTs during the larval period, when these enzymes are important in managing the potential toxicity from host plant secondary metabolites. This role seems particularly well-suited for caterpillars, or ‘eating machines’ [94], which are specialized in mass accumulation and energy storage for subsequent developmental stages.

The increased LDH activity observed during the larval-pupal transition in our study suggests a shift towards anaerobic metabolism. This is consistent with similar findings in the early pupal stages of the fly *Calliphora erythrocephala* [70,78] and other organisms experiencing oxygen scarcity, either from limited environmental oxygen availability [95] or from an imbalance between oxygen demand and supply, as in intense exercise [96]. Additionally, our results align with the hypothesis that the reorganization of the tracheal network during complete metamorphosis [97,98,99,100] may limit oxygen supply to the pupal tissues [8]. Other indirect evidence of hypoxia includes redox imbalance, oxidative stress and upregulation of antioxidants. These biochemical events occurred in the early pupal stage of *C. lacinia* and are associated with exposure to hypoxia or conditions that impair proper oxygen delivery in a wide range of animals [27,29]. For example, both hypoxia-sensitive (such as mice [101]) and hypoxia-tolerant species [102] experience redox imbalance and oxidative stress in response to hypoxia [29]. Moreover, the upregulation of endogenous antioxidants is a hallmark of hypoxia-tolerant species [103], a phenomenon known as ‘Preparation for Oxidative Stress’ [28,29,104]. While several pieces of evidence from our study suggest a period of oxygen limitation during pupation, the lack of direct measurement of pO_2_ availability or anaerobic end-products in our study precludes a definitive confirmation of a hypothetical oxygen limitation at this stage. To the best of our knowledge, only one study has directly addressed the occurrence of hypoxia in early pupa and found no evidence for oxygen limitation in *D. melanogaster* [8].

The elevation in LDH activity has implications for redox metabolism other than its role in glycolysis. LDH can influence the redox state of cells, exhibiting either antioxidant or pro-oxidant effects depending on the context [105,106]. LDH can catalyze a chain reaction initiated and propagated by superoxide radicals, leading to hydrogen peroxide production [107,108]. The physiological relevance of these reactions has been investigated and, for instance, downregulating LDH in cancer cell lines and primary cultures leads to decreased ROS production [105,109]. Here, increased LDH activity during the larval-pupal transition correlated with glutathione depletion, altered redox balance, increased lipid peroxidation, and activation of GPX. Given that superoxide production gradually increases as larvae of *B. mori* approaches pupation [53], the potential pro-oxidant effect of LDH is expected to be boosted at this development stage. However, the precise link between LDH activity and redox balance disruption in complete insect metamorphosis requires further exploration.

Our findings are consistent with previous observations indicating an association between oxidative stress and pupation in holometabolous insects. In *T. molitor*, pupae have the highest level of lipid peroxidation, measured as conjugated dienes, when compared with larvae and imagoes [46]. Similarly, early pupa of *G. mellonella* have higher TBARS levels than its preceding developmental stages [52]. Oxidative stress in early pupae is consistent with the progressive decline in SOD1 and SOD2 protein levels, alongside rising superoxide levels in *B. mori* from day three of the fifth instar until pupation [53]. The downregulation of several SOD genes in *B. mori* pupae relative to larval stages reinforces this trend [43]. Biophoton emission imaging suggests a surge in the production of excited molecules, such as RS, during the first two hours post-ecdysis in pupae of the spangle, *Papilio protenor* [110]. These findings collectively indicate that pupation represents a critical period of disturbed redox balance and oxidative stress.

Deviations from the physiological ‘redox set point’ trigger compensatory adjustments in antioxidant systems [20]. While previous studies had already identified an increase in non-specific peroxidase activity in early pupae versus larvae of *D. melanogaster* [55,111], here we identified glutathione peroxidases as one of the sources of upregulated capacity to decompose peroxides in this period. Noteworthily, this was accompanied by an increased GSSG/tGSH ratio, which could be a consequence of increased GPX activity. Our findings further compose the redox landscape during pupation, showing that glutathione depletion and GPX activation occurs at this same time point. The modulation of SOD expression and activity by ecdysone injection and the suppression of pupation by the administration of a SOD mimic demonstrate the key participation of redox adjustments in insect development and metamorphosis [53]. Indeed, antioxidant systems have been implicated in insect metamorphosis and deviations from direct development (i.e., diapause). For example, caterpillars of *C. lacinia* increase their GST activity and NADPH-producing capacity during the first hours of entering diapause [32], and antioxidant genes are upregulated in the brain of the cotton bollworm *Helicoverpa armigera* at diapause initiation [112]. Our present study further points to a possible role of glutathione dependent systems in redox events during pupation, as observed in zebrafish embryonic development [113], which warrants further research in holometabolous insects.

Our study presents valuable insights into redox metabolism during holometabolous metamorphosis by focusing on a lepidopteran species with remarkable resilience to environmental stressors. While our approach proved insightful, investigating other Endopterygota species is necessary to address the generalizability of our findings and enhance our understanding of biochemical adjustments in redox metabolism during complete insect metamorphosis. Additionally, the relatively small sample size used in our study may limit the robustness of our conclusions. Nevertheless, this study serves as a starting point for further research to explore causal relationships between shifts in redox metabolism and successful complete metamorphosis in insects. For instance, based on the pathways modulated in our study, future research manipulating specific biochemical pathways can clarify how changes in redox metabolism might affect complete metamorphosis not only under optimal conditions but also when exposed to environmental stressors such as pollutants, varying temperatures, and humidity.

## 5. Conclusions

This study set out to gain a better understanding of the biochemical changes in metabolism, antioxidant systems, and redox balance that occur during complete insect metamorphosis. Studying the sunflower caterpillar *Chlosyne lacinia*, we found that the larva-to-pupa transition is a period of oxidative eustress, characterized by simultaneous increased anaerobic capacity, redox imbalance, oxidative damage and activation of an antioxidant enzyme. As metamorphosis progressed, a return to a more reduced state is observed, indicative of effective biochemical adaptations countering this initial oxidative stress. The adult emergence phase is marked by an increase in oxidative metabolism, which poses additional oxidative challenges. These are managed by enhanced antioxidant systems. Beyond corroborating known aspects of redox metabolism, our study uncovers previously unidentified events and molecular players in redox metabolism during complete insect metamorphosis. These findings not only extend our knowledge of redox metabolism adjustments, but also pave the way for future research into the redox biology of complete insect metamorphosis.

## Data Availability

The original contributions presented in the study are included in the article and Supplementary Materials; further inquiries can be directed to the corresponding author.

## Supplementary Materials

The following supporting information can be downloaded at: https://www.mdpi.com/article/10.3390/antiox13080959/s1.
